# *RAD51B^me^* Levels as a Potential Predictive Biomarker for PD-1 Blockade Response in Non-Small Cell Lung Cancer

**DOI:** 10.3390/jcm9041000

**Published:** 2020-04-02

**Authors:** Inês Maria Guerreiro, Daniela Barros-Silva, Paula Lopes, Mariana Cantante, Ana Luísa Cunha, João Lobo, Luís Antunes, Ana Rodrigues, Marta Soares, Rui Henrique, Carmen Jerónimo

**Affiliations:** 1 Department of Medical Oncology, Portuguese Oncology Institute of Porto (IPO-Porto), R. Dr. António Bernardino de Almeida, 4200-072 Porto, Portugal; rodriguesana@me.com (A.R.); martasoares71@gmail.com (M.S.); 2 Cancer Biology and Epigenetics Group, IPO Porto Research Center (GEBC CI-IPOP), Portuguese Oncology Institute of Porto (IPO Porto) & Porto Comprehensive Cancer Center (P.CCC), R. Dr. António Bernardino de Almeida, 4200-072 Porto, Portugal; daniela.barros.silva94@gmail.com (D.B.-S.); lopesanapaula.s@gmail.com (P.L.); marianacantantecf@gmail.com (M.C.); analuisa.cunha@ipoporto.min-saude.pt (A.L.C.); jpedro.lobo@ipoporto.min-saude.pt (J.L.); henrique@ipoporto.min-saude.pt (R.H.); 3 Department of Pathology, Portuguese Oncology Institute of Porto (IPOP), R. Dr. António Bernardino de Almeida, 4200-072 Porto, Portugal; 4 Department of Pathology and Molecular Immunology, Institute of Biomedical Sciences Abel Salazar, University of Porto (ICBAS-UP), Rua Jorge de Viterbo Ferreira, 228, 4050-313 Porto, Portugal; 5 Cancer Epidemiology Group, IPO Porto Research Center (CI-IPOP), Portuguese Oncology Institute of Porto (IPO-Porto), R. Dr. António Bernardino de Almeida, 4200-072 Porto, Portugal; luis.antunes@ipoporto.min-saude.pt

**Keywords:** *RAD51B* methylation, PD-L1 expression, predictive biomarker, PD-1 blockade

## Abstract

Lung cancer (LC) cells frequently express high levels of programmed death-ligand 1 (PD-L1). Although these levels grossly correlate with the likelihood of response to specific checkpoint inhibitors, the response prediction is rather imperfect, and more accurate predictive biomarkers are mandatory. We examined the methylation profile of *RAD51B* (*RAD51B^me^*) as a candidate predictive biomarker for anti-PD-1 therapy efficacy in non-small cell lung cancer (NSCLC), correlating with patients’ outcome. PD-L1 immunoexpression and *RAD51B^me^* levels were analysed in NSCLC samples obtained from patients not treated with anti-PD-1 (Untreated Cohort (#1)) and patients treated with PD-1 blockade (Treated Cohort (#2)). Of a total of 127 patients assessed, 58.3% depicted PD-L1 positivity (PD-L1^+^). *RAD51B^me^* levels were significantly associated with PD-L1 immunoexpression. Patients with PD-1 blockade clinical benefit disclosed higher *RAD51B^me^* levels (*p* = 0.0390) and significantly lower risk of disease progression (HR 0.37; 95% CI: 0.15–0.88; *p* = 0.025). Combining *RAD51B^me+^* with PD-L1^+^ improved the sensitivity of the test to predict immunotherapy response. PD-L1^+^ was also associated with lower risk of death (HR 0.35; 95% CI: 0.15–0.81; *p* = 0.014). Thus, *RAD51B^me^* levels might be combined with validated predictive biomarker PD-L1 immunostaining to select patients who will most likely experience clinical benefit from PD-1 blockade. The predictive value of *RAD51B^me^* should be confirmed in prospective studies.

## 1. Introduction

Lung cancer is the leading cause of cancer death in Europe, with an estimated 470,000 new cases (311,000 in men and 158,200 in women) in 2018 [1]. The estimated mortality in 2018 was 20.1% in both genders, being the most common cause of death from cancer in men (267,000 deaths, 24.8%) and the second most frequent in women (121,000 deaths, 14.2%) [1]. Most patients are diagnosed at advanced stages, with an overall 5-year survival rate of 4–17% depending on the stage and regional differences [2]. The incidence of lung cancer is directly related to tobacco smoking, which is the primary cause of lung cancer, accounting for about 80% to 90% of cases [3]. The risk of lung cancer increases with the extent of smoking measured by the number of packs of cigarettes smoked per day and with the number of years of smoking (pack-years of smoking history) [4].

Since the emergence of personalised targeted therapies, pathology plays a critical role because histologic and genetic features of lung cancer are important determinants of molecular testing and treatment decisions [5,6,7]. Lung cancer can be classified in non-small cell lung cancer (NSCLC) and small-cell lung cancer [5]. NSCLC is the most frequent class of lung cancer, representing 80% of all cases [4] and includes non-squamous carcinoma and squamous cell carcinoma as major types [5]. Non-squamous carcinoma includes adenocarcinoma, which is the most common subtype of lung cancer [4]. When clear adenocarcinoma, squamous or neuroendocrine morphology or staining pattern is not present, NSCLC is generally classified as not otherwise specified (NOS) [5].

Several predictive biomarkers indicative of therapeutic efficacy have emerged in lung cancer [6]. Immunotherapy, mainly immune checkpoint inhibitors, has changed the treatment paradigm of NSCLC. Immune checkpoints are important to control the immune responses in order to protect tissues from damage when the immune system is activated [8]. The expression of immune checkpoint proteins can be dysregulated by cancer cells, enabling immune evasion, a cancer hallmark [8,9]. Programmed cell death protein 1 (PD-1) is an immune checkpoint receptor expressed on the surface of activated T cells, including a large proportion of tumour-infiltrating lymphocytes from many tumours [8,10]. The binding to its ligands, PD-L1 and PD-L2, inhibits the response of cytotoxic T cells, hence the activation of the pathway PD-1/PD-L1 is a mechanism of immune-escape [11]. PD-L1 is commonly upregulated at the tumour cell surface [8] and is generally expressed in 20% to 40% of NSCLC [12]. There is evidence that infiltrating lymphocytes, mutational burden, and the expression of PD-L1 [13,14] are predictive biomarkers for treatment with checkpoint inhibitors. However, prediction of response is rather imperfect and, thus, more accurate predictive biomarkers are mandatory.

Genome instability leading to the accumulation of genomic aberrations is another characteristic of cancer cells [9]. Double-strand DNA breaks (DSB) may lead to mutations, chromosomal translocations, cell senescence and apoptosis [15,16]; hence, repair mechanisms are essential to maintain genome stability. Homologous recombination repair (HRR) is the leading DNA repair mechanism of double-strand DNA breaks (DSB) that uses the homologous region of the sister chromatid as the replicative template in order to reliably repair DSB [16]. *RAD51* protein has an important activity in HRR, promoting the insertion of the broken ends of the DSB into the sister chromatid [17,18]. Its action is dependent on *RAD51*-like proteins: *RAD51B*, *RAD51C*, *RAD51D*, *XRCC2* and *XRCC3* [17,18,19]. Defects in the HRR pathway entail cell proliferation despite DNA damage, promoting cancer development [20]. HRR pathway deficiencies seem to be associated with higher expression of PD-L1 and linked to an immune-evasive tumour phenotype [16]. Rieke et al. found that HRR genes hypermethylation is inversely correlated with mRNA transcription and associated with PD-L1 expression in head and neck, lung, and cervix squamous cell carcinomas [18]. As such, the methylation status of these genes could represent new predictive biomarkers for immune checkpoint inhibition.

The aim of this study is to investigate the association of immune checkpoint PD-L1 expression and the status of DNA repair gene *RAD51B* promoter methylation (*RAD51B^me^*) in advanced NSCLC, correlating with patients’ outcome. Additionally, the potential of *RAD51B^me^* levels as a candidate predictive biomarker for PD-1 blockade response in NSCLC was also assessed.

## 2. Materials and Methods

### 2.1. Patient Selection

We retrospectively analysed patients ≥18 years old, diagnosed with advanced NSCLC (adenocarcinoma, squamous cell carcinoma, and non-small cell lung cancer, not otherwise specified), at the Portuguese Oncology Institute of Porto (IPO-Porto) between 2014 and 2019. All tissue samples were obtained at the time of diagnosis. Samples were routinely fixed, and paraffin-embedded for standard pathological examination by haematoxylin and eosin (H&E) and specific immunostaining for tumour classification, grading, and staging, according to World Health Organization (WHO) Classification of Tumours of the Lung, Pleura, Thymus and Heart (4th Edition, Volume 7). Specimens were evaluated by two lung pathology proficient pathologists (ALC and RH). Biopsy samples available at the archive of the Department of Pathology were obtained for the “Untreated” cohort (Cohort #1, patients not exposed to anti-PD-1 blockade) and “Treated” cohort (Cohort #2, patients exposed to anti-PD-1 blockade anytime during the course of the disease) and were included after approval by the ethics committee of IPO-Porto (CES 15R1/2017).

### 2.2. Clinical and Pathological Data Collection

Relevant clinical and pathological variables were retrospectively collected for patients’ characterisation, including pathological diagnosis (adenocarcinoma, squamous cell carcinoma, not otherwise specified), gender (female, male), age, smoking habits (never smoker, smoker, previous smoker), stage of the disease (stages IIIA to IVB were considered as advanced disease) and type of anti-PD-1 treatment (nivolumab, pembrolizumab, according to the current practice at the time).

All patients whose tumours displayed ≥50% PD-L1 expression did pembrolizumab as a first-line treatment [21], patients whose tumours had 1–49% PD-L1 expression did pembrolizumab [22] or nivolumab as second line treatment, and those with negative PD-L1 expression did nivolumab as a second-line treatment after progression of disease on or after standard platinum-based chemotherapy [23,24]. In patients whose tumours presented a driver mutation (epidermal growth factor receptor (*EGFR*) tyrosine kinase mutation, anaplastic lymphoma kinase (*ALK*) gene rearrangement or c-ROS oncogene 1 (*ROS1*) translocations), treatment with anti-PD-1 was done after progression on or after tyrosine kinase inhibitors and platinum-based chemotherapy.

Response to treatment was assessed by using the Response Evaluation Criteria in Solid Tumours (RECIST): complete response (CR)—disappearance of all target lesions, pathological lymph nodes must have reduction in short axis to <10 mm; partial response (PR)—at least a 30% decrease in the sum of diameters of target lesions, taking as reference the baseline sum diameters; progressive disease (PD)—at least a 20% increase in the sum of diameters of target lesions, taking as reference the smallest sum on study which must demonstrate an absolute increase of at least 5 mm; stable disease (SD)—neither sufficient shrinkage to qualify for PR nor sufficient increase to qualify for PD. Clinical benefit was considered if CR, PR or SD were present.

All procedures performed were in accordance with the ethical standards of the institutional and national research committees and with the 1964 Helsinki declaration and its later amendments or comparable ethical standards.

### 2.3. Assessment of PD-L1 Expression by Immunohistochemistry

PD-L1 (dilution 1:100, clone 22C3, DAKO) immunostaining was performed on a BenchMark Ultra platform (Ventana, Tucson, AZ, USA) using OptiView DAB detection kit (Ventana, Tucson, AZ, USA) and high pH buffer solution (CC1, Ventana, Tucson, AZ, USA for 40 min at 95 °C) was used for antigen retrieval. Appropriate positive controls were used for each antibody and negative controls consisted of omission of primary antibody. PD-L1 expression was assessed by a proficient pathologist (ALC) who determined the tumour proportion score (TPS), according to the European Society for Medical Oncology (ESMO) guidelines. TPS was considered negative if <1%, positive intermediate if 1–49%, and positive strong if ≥50%.

### 2.4. Methylation Analysis

DNA and RNA were extracted from all clinical samples and cell lines using an FFPE RNA/DNA Purification Plus Kit (Norgen, Thorold, ON, Canada), according to the manufacturer’s instructions. The bisulfide modification was accomplished using an EZ DNA Methylation-Gold™ Kit (Zymo Research, Orange, CA, USA) that integrates DNA denaturation and the bisulfide conversion processes into one-step, according to the recommended protocol. Evaluation of the DNA repair genes’ methylation status was done by quantitative methylation-specific PCR (qMSP) assays and was performed using Xpert Fast SYBR (GRiSP, Porto, Portugal), according to the recommended protocol, in 384-well plates using a Roche LightCycler 480 II. Primers addressing the informative CpG sites within the promoter region were designed using Methyl Primer Express v1 and are described in Table 1. *β-actin* (ACTB) was used as an internal reference gene for normalization.

### 2.5. Statistical Analysis

Statistical analysis was conducted separately for each cohort.

Categorical variables are presented as counts and proportions and continuous variables are displayed as mean (standard deviation). Median (interquartile range) is used to describe variables with a highly skewed distribution.

Chi-square test was used to test the association between categorical variables; the Mann–Whitney U test was used to compare continuous variables with skewed distribution. A logistic regression analysis was carried out to identify predictors of PD-L1 expression. The variables considered in the logistic regression model were *RAD51B^me^* (continuous), sex, age, smoking status and histological subtype.

The area under the receiver operating characteristics curve (AUC, 95% CI) was analysed to assess the performance of the *RAD51B* promotor methylation level as a predictive biomarker for PD-1 blockade response. Specificity, sensitivity, positive predictive value (PPV), negative predictive value (NPV), and accuracy were determined for PD-L1, according to positive vs. negative immune scores and for *RAD51B* methylation by applying an empirical cut-off obtained by ROC curve analysis (sensitivity + (1-specificity)). This cut-off value combines the maximum sensitivity and specificity, ensuring the perfect categorization of the samples as positive and negative for the methylation test. For the analysis of combined *RAD51B^me+^*/PD-L1^+^, the test was considered positive when at least one of the variables was plotted, as positive in individual analysis. Diagnostic biomarker performance was calculated, taking into consideration that all the patients included were subjected to anti-PD-1 treatment.

Progression-free survival (PFS) and overall survival (OS) were estimated by means of the Kaplan–Meier method for the Treated Cohort (#2). PFS was defined as the length of time from the beginning of anti-PD-1 blockade until disease progression or death from the disease and OS as the length of time from the beginning of anti-PD-1 blockade until death from any cause. The differences between groups were tested using the log-rank test. Hazard ratios (HRs) from multivariable Cox regression were used to quantify the association between clinicopathological features and survival. *RAD51B* promoter methylation level was considered positive if the quantitative value was above the 75^th^ percentile. A *p*-value smaller than 0.05 (two-sided) indicated statistical significance.

All analyses were performed using IBM SPSS Statistics version 26.0 (SPSS, Chicago, IL, USA) and GraphPad Prism 7.01 (GraphPad Software, La Jolla, CA, USA).

## 3. Results

Between 2014 and 2019, 293 patients fulfilling the inclusion criteria were analysed. The median age was 64 years, 79.9% were male, and most of the patients (70%) presented adenocarcinoma. A biopsy sample was available in 127 (43.3%) patients *(n* = 64 in Untreated Cohort (#1) and *n* = 63 in Treated Cohort (#2)). PD-L1 expression was deemed positive in 58.3% cases *(n* = 31 in Untreated Cohort (#1) and *n* = 43 in Treated Cohort (#2)). Table 2 depicts patients’ characteristics in the Untreated and Treated cohorts.

In the Treated Cohort (#2), 18 patients whose tumours showed ≥50% PD-L1 expression were treated with pembrolizumab in first-line; 19 and 3 patients whose tumours had 1–49% PD-L1 expression were treated with pembrolizumab and nivolumab, respectively, in second-line after progression on chemotherapy. Eighteen patients with PD-L1 negative tumours were treated with nivolumab as a second-line treatment. Four patients with adenocarcinoma carried driver mutations (3 had an *EGFR* tyrosine kinase mutation and 1 had an *ALK* gene rearrangement). As such, anti-PD-1 therapy was administered as a third-line treatment, after progression on tyrosine kinase inhibitors (first-line) and chemotherapy (second-line).

**Figure 1:**
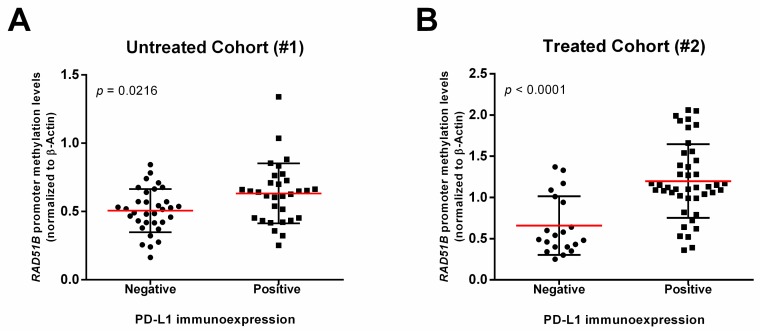
*RAD51B* promoter methylation levels within PD-L1 negative and PD-L1 positive immunoexpression among NSCLC samples. Scatter plot representing *RAD51B* promoter methylation levels distribution obtained by qMSP for (**A**) Untreated Cohort (#1) and (**B**) Treated Cohort (#2) patients, according to negative and positive PD-L1 immunoexpression. Mann–Whitney U-test. Red horizontal line represents the median methylation levels.

Regarding molecular analysis, *RAD51B^me^* levels were significantly higher in PD-L1 positive vs. negative cases in both cohorts (Untreated Cohort (#1)—*p* = 0.0216; Treated Cohort (#2)—*p* < 0.0001) (Figure 1). Patients presenting higher *RAD51B^me^* levels showed a higher chance of having a positive PD-L1 immunoexpression (Untreated cohort (#1) OR: 51.68, 95% CI: 1.77–1512.04, *p* = 0.022; Treated cohort (#2) OR: 45.51, 95% CI: 5.29–391.20, *p* = 0.001), adjusting for sex, age, smoking status and histological subtype (detailed information in Table S1). No differences in *RAD51B^me^* levels were found between squamous cell carcinoma and adenocarcinoma cases in both cohorts (Untreated Cohort (#1)—*p* = 0.774; Treated Cohort (#2)—*p* = 0.520).

**Figure 2:**
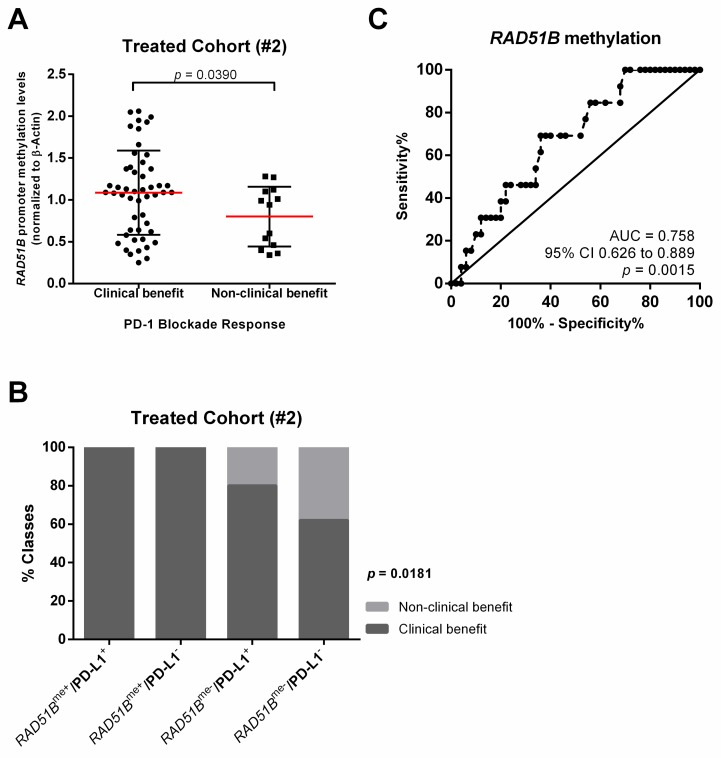
*RAD51B^me^* levels and PD-L1 positivity associate with PD-1 blockade clinical benefit. (**A**) Scatter plot representing *RAD51B* promoter methylation levels distribution obtained by qMSP in patients with and without clinical benefit from immunotherapy. Mann–Whitney U-test. Red horizontal line represents the median methylation levels; (**B**) Contingency graph displaying the percentage of patients with and without PD-1 blockade clinical benefit, according to *RAD51B* promoter methylation and PD-L1 status. Chi-square test. *RAD51B^me^* were considered positive when promoter methylation levels >P75; (**C**) Receiver operator characteristic (ROC) curve for discrimination between patients with and without clinical benefit from immunotherapy based on *RAD51B* promoter methylation levels distribution in the Treated Cohort (#2).

*RAD51B^me^* levels were significantly higher in patients submitted to immunotherapy, which demonstrated clinical benefit (*p* = 0.0390; Figure 2A). Moreover, patients with positive *RAD51B^me^* levels (*RAD51B^me+^* was consider when methylation levels >P75) disclosed clinical benefit independently from PD-L1 expression (Figure 2B). Additionally, *RAD51B^me^* discriminated between PD-1 blockade clinical benefit and no clinical benefit with 85% specificity and 90% positive predictive value (AUC: 0.758, 95% CI: 0.626–0.889, *p* = 0.0015; Figure 2C and Table 3). Remarkably, combining *RAD51B^me+^* with PD-L1^+^ improved the sensitivity of the test (68%) to predict immunotherapy response, maintaining high specificity (85%) and increasing positive predictive value (94%).

**Figure 3:**
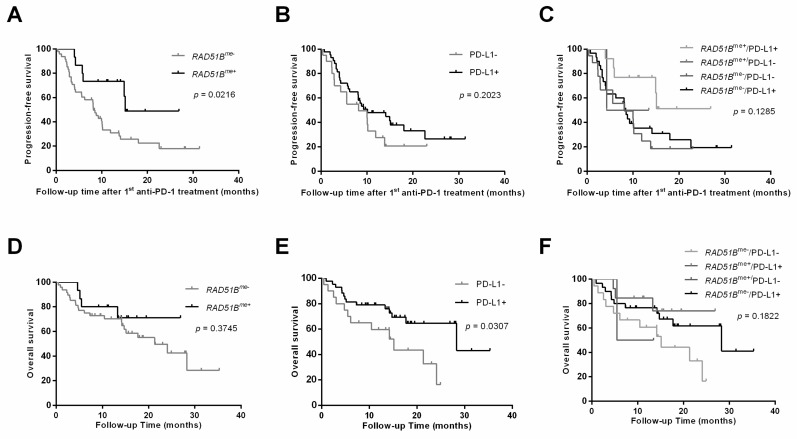
Kaplan–Meier survival curves for progression-free survival (after first anti-PD-1 treatment) of patients according to (**A**) *RAD51B^me^* status; (**B**) PD-L1 status; and (**C**) combined *RAD51B^me^* and PD-L1 status. Kaplan–Meier survival curves for patients’ overall survival according to (**D**) *RAD51B^me^* status, (**E**) PD-L1 status, and (**F**) combined *RAD51B^me^* and PD-L1 status. Log-rank test. *RAD51B^me^* was considered positive when promoter methylation levels >P75.

The median follow-up time for the Treated Cohort (#2) was 18 months (95% CI: 15.1–20.9). The median PFS was significantly higher in *RAD51B^me+^* patients (*p* = 0.0216; Figure 3A). Furthermore, patients with *RAD51B^me+^* disclosed a lower risk of disease progression (HR 0.37; 95% CI: 0.15–0.88; *p* = 0.025) compared with *RAD51B^me-^*. Considering the PD-L1 expression, no significant differences were depicted for PFS (*p* = 0.2023), although PD-L1^+^ patients disclosed a trend for higher PFS (Figure 3B). Nonetheless, PD-L1^+^ associated with a longer OS (*p* = 0.0307) and a lower risk of death (HR 0.35; 95% CI: 0.15–0.81; *p* = 0.014). For *RAD51B*, lower methylation levels tend to associate with shorter OS, despite not being statistically significant. Also, no significant differences were observed for PFS or OS, when combining in panel PD-L1 expression and *RAD51B^me^* levels.

## 4. Discussion

Despite the improvement in lung cancer treatment over the last years, it remains a lethal disease in most cases, mostly due to diagnosis at advanced stages and suboptimal effectiveness of standard therapy. Nonetheless, the emergence of novel therapeutic strategies, including immune-based cancer therapies, has improved the prospects of patients diagnosed at advanced stages of the disease. Indeed, anti-PD-1 treatment for advanced NSCLC has improved the survival of patients [22]. Currently, the most commonly used biomarker to predict this response to anti-PD-1 therapy is PD-L1 immunostaining, although a substantial number of patients with PD-L1 positive immunostaining do not respond [21], highlighting the need for new biomarkers. In NSCLC, similar to other tumours, a higher tumour mutation burden was a strong predictor of immunotherapy efficacy [25,26,27,28]. Additionally, defects in the HRR pathway have been associated with higher expression of co-regulatory molecules such as PD-L1, suggesting that deficient homologous recombination, by disabling repair of DNA defects, may lead to neoantigens production with the recruitment of T-cells to the tumour microenvironment. This engages tumour cells to upregulate the expression of PD-L1 as an adaptive resistance mechanism [29]. A recent study demonstrated that DNA methylation profile of NSCLC might also be determinant for the efficacy of anti-PD-1 treatment in stage IV patients [30]. Furthermore, epigenetic alterations in *RAD51B*, specifically DNA promoter methylation, were associated with PD-L1 expression in squamous cell carcinomas [18]. This is a *RAD51* paralog, essential for DSB repair in the homologous recombinant pathway [17]. Thus, we sought to investigate the association of immune checkpoint PD-L1 expression and DNA methylation status of DNA repair gene *RAD51B* in non-small cell lung cancer (NSCLC), correlating with patient outcome.

Overall, the chances of positive PD-L1 expression in advanced NSCLC increased with the level of *RAD51^me+^*. Remarkably, a link between *RAD51B^me^* and the immune response in NSCLC has been previously suggested [29]. Furthermore, *Rieke et al.* demonstrated that methylation was associated with low mRNA expression levels and with homologous recombination deficiency [18]. Additionally, a significant positive correlation between *RAD51B* methylation status and the inflammatory gene signature, particularly, interferon-gamma (IFN-γ) was disclosed [18]. Interestingly, IFN-γ is an important inducer of PD-L1 expression, which acts via the JAK/STAT1/interferon regulatory factor (IRF) [31] in various types of cancers, including NSCLC. Furthermore, the depletion of *RAD51B* was shown to induce immune response through activation of the STAT3 pathway [32], which activates *CD274* gene/PD-L1 induction [31,33]. Therefore, our results further support the link between homologous repair deficiency by epigenetic regulation and immune checkpoint players, specifically PD-L1. Considering the available literature, assessing the inflammatory profile of these tumours might be useful to determine whether there is a direct effect between DNA repair candidate genes hypermethylation and the expression of immune checkpoint proteins.

Remarkably, *RAD51B^me+^* associated with better clinical response to treatment with PD-1 blockade and to a reduction of disease progression by 60%. Conversely, *RAD51B^me-^* associated with the absence of clinical benefit, which was even more relevant in negative PD-L1 expression cases. Hence, *RAD51B^me^* might constitute a potential biomarker of response to anti-PD-1 therapy. Although *RAD51B^me^* depicted lower sensitivity than PD-L1^+^ as a predictive biomarker for treatment with anti-PD-1, it displayed higher specificity.

Although PD-L1 expression has not been described as a strong prognostic factor mostly due to methodological approaches variations, including diverse immunohistochemistry antibodies, dissimilar evaluation for PD-L1 positivity (cut-off % or H-score) and patients’ selection [13,34], in our study, both PD-L1^+^ and *RAD51B^me+^* associated with better overall survival. Conversely, another research team suggested that *RAD51B* overexpression associates with improved OS in NSCLC patients [35]. Notwithstanding higher promoter methylation levels might entail expression downregulation, several other genetic and epigenetic mechanisms may contribute to this apparent inconsistency. Furthermore, higher *RAD51B* methylation status was depicted in patients with longer progression-free survival after anti-PD-1 treatment, supporting once more the clinical benefit of PD-1 blockade when *RAD51B* promoter is methylated. The shorter overall survival of non-smokers patients may be partially explained by the fact that these patients had a longer median time (higher than 20 months) between diagnosis and the treatment with PD-L1 inhibitors than smokers.

Therefore, PD-L1^+^ and *RAD51B^me+^* are promising biomarkers to predict response to PD-1 blockade rather than overall prognostic factors in NSCLC’s patients. As such, *RAD51B^me^* might represent a new predictive marker potentially assessable in liquid biopsies, allowing for a better selection of patients for anti-PD-1 treatment and eventually for monitoring patients’ immunotherapy response throughout the course of the disease. Although our study paves the way for new prospective studies on the *RAD51B* promoter methylation’s predictive role in patients with NSCLC treated with anti-PD-1, the retrospective design and small sample size are not neglectable limitations. Nevertheless, all the patients and samples enrolled in the study were analysed using the same criteria both for molecular biology strategies or clinical and pathological data collection. Importantly, other strengths of our research work are the fact that all patients were uniformly treated at the same institution, and all were evaluated by computed tomographic scans at specific timepoints during the course of treatment.

## 5. Conclusions

Herein, we confirm that higher *RAD51B^me^* levels associate with PD-L1 immunoexpression, as well as with immunotherapy’s efficacy, in an independent advanced NSCLC patient cohort. Prospective studies, with larger cohorts of patients and extended follow-up periods, are warranted to validate these results and determine whether the methylation profile of this gene might be a predictive tool for selecting patients that will benefit from anti-PD-1 therapy.

## Figures and Tables

**Table 1:** Primer sequences for *ß-Actin* and *RAD51B^me^*.

Gene	Forward (5′–3′)	Reverse (5′–3′)
*ß-Actin*	TGGTGATGGAGGAGGTTTAGTAAGT	AACCAATAAAACCTACTCCTCCCTTAA
*RAD51B^me^*	AGATTTTTAGGGTCGAGAGC	CGCCCGACTAATTTTTTTAT

**Table 2:** Clinical and pathological data according to the testing cohorts.

Characteristics	Untreated Cohort (#1) *n* = 64	Treated Cohort (#2) *n* = 63
Gender, (*n*, %) Male Female	51 (79.7) 13 (20.3)	49 (77.8) 14 (22.2)
Age (year), median (IQR)	62.5 (29.0–84.0)	62.0 (32.0–77.0)
Histologic subtype (*n*, %) Adenocarcinoma Squamous NOS	41 (64.1) 22 (34.4) 1 (1.6)	46 (73.0) 17 (27.0) -
Smoking habits (*n*, %) Never Smoker Previous smoker	16 (25.0) 20 (31.3) 28 (43.7)	10 (15.9) 20 (31.7) 33 (53.4)
PD-L1 immunoexpression (*n*, %) Negative Intermediate (1–49%) Strong (≥ 50%)	33 (51.6) 18 (28.1) 13 (20.3)	20 (31.7) 14 (22.2) 29 (46.0)
Anti-PD-1 agent (*n*, %) Pembrolizumab Nivolumab	n.a.	38 (60.3) 25 (39.7)
PD-1 blockade (*n*, %) Clinical benefit Non-clinical benefit	n.a.	13 (20.6) 50 (79.4)
End of PD-1 blockade treatment (*n*, %) Not applicable Disease progression Toxicity	n.a.	18 (28.6) 39 (61.9) 6 (9.5)
Progression-free survival since PD-1 blockade, months median (IQR)	n.a.	8.1 (5.1–11.1)
Overall survival since PD-1 blockade, months median (IQR)	n.a.	21.3 (13.7–28.9)
*RAD51B^me^* levels (normalized to β-actin), median (IQR)	0.54 (0.16–1.34)	1.08 (0.25–2.06)

n.a.—not applicable; IQR – Interquartil Range.

**Table 3:** *RAD51B^me^*, PD-L1 staining and the combination of the two variables performances as predictive biomarkers of PD-1 blockade response in the Treated Cohort (#2).

Predictive Biomarkers of PD-1 Blockade Response			
	***RAD51B^me+^***	**PD-L1^+^**	***RAD51B^me+^*/PD-L1^+^**
Sensitivity	38%	74%	68%
Specificity	85%	54%	85%
Accuracy	48%	70%	71%
PPV	90%	86%	94%
NPV	26%	35%	41%

Abbreviations: PPV: positive predictive value, NPV: negative predictive value.

## Supplementary Materials

The following are available online at https://www.mdpi.com/2077-0383/9/4/1000/s1. Table S1: Logistic regression analysis; Table S2: Multivariable analysis for progression-free survival; Table S3: Multivariable analysis for overall survival.
